# Unraveling the rate-limiting step of two-electron transfer electrochemical reduction of carbon dioxide

**DOI:** 10.1038/s41467-022-28436-z

**Published:** 2022-02-10

**Authors:** Wanyu Deng, Peng Zhang, Brian Seger, Jinlong Gong

**Affiliations:** 1 Key Laboratory for Green Chemical Technology of Ministry of Education, School of Chemical Engineering and Technology, Tianjin University, Collaborative Innovation Center of Chemical Science and Engineering (Tianjin), Tianjin, 300072 China; 2 SurfCat, Department of Physics, Technical University of Denmark, 2800 Kgs Lyngby, Denmark; 3 Joint School of National University of Singapore and Tianjin University, International Campus of Tianjin University, Binhai New City, Fuzhou, 350207 China

**Keywords:** Electrocatalysis, Energy

## Abstract

Electrochemical reduction of CO_2_ (CO_2_ER) has received significant attention due to its potential to sustainably produce valuable fuels and chemicals. However, the reaction mechanism is still not well understood. One vital debate is whether the rate-limiting step (RLS) is dominated by the availability of protons, the conversion of water molecules, or the adsorption of CO_2_. This paper describes insights into the RLS by investigating pH dependency and kinetic isotope effect with respect to the rate expression of CO_2_ER. Focusing on electrocatalysts geared towards two-electron transfer reactions, we find the generation rates of CO and formate to be invariant with either pH or deuteration of the electrolyte over Au, Ag, Sn, and In. We elucidate the RLS of two-electron transfer CO_2_ER to be the adsorption of CO_2_ onto the surface of electrocatalysts. We expect this finding to provide guidance for improving CO_2_ER activity through the enhancement of the CO_2_ adsorption processes by strategies such as surface modification of catalysts as well as careful control of pressure and interfacial electric field within reactors.

## Abstract (web summary)

Electroreduction of CO_2_ is heavily investigated but its reaction mechanism needs to be further explored. Here, the authors investigate pH dependency and kinetic isotope effect with respect to the rate expression of CO_2_ electroreduction to gain further insights into the rate-limiting step.

## Introduction

In order to alleviate greenhouse effects, countries around the world have formulated different carbon neutralization plans^1^. Electrochemical reduction of CO_2_ (CO_2_ER) can convert CO_2_ into valuable fuels and chemicals^2–7^, which is an emerging approach to utilize CO_2_ as a resource. CO_2_ER to either CO or formate through the two-electron transfer reaction is promising for industrialization due to its high selectivity and high current density^8,9^. An in-depth understanding of the two-electron transfer reaction mechanisms is, therefore, essential to designing high-efficiency catalysts and advancing the progress of CO_2_ER towards commercialization.

Recently, the two-electron transfer of CO_2_ER to formate and CO has been widely accepted to proceed through the pathways with intermediate species of *OOCH and *COOH, respectively^10,11^, as inner-sphere electron-transfer reactions^12,13^. However, there is still a debate in terms of the RLS^14–16^. Specifically, the RLS has been considered to be the adsorption of CO_2_ along with an electron transfer over the catalysts^17–21^, the transfer of protons to *CO_2_^−^ to form *COOH or *OOCH^16,22,23^, the proton-coupled electron transfer to form *COOH or *OOCH^24^, or the desorption of *CO^25,26^ according to the results of Tafel analysis^16,17,22^, reaction order analysis^20,21^, and theoretical calculations^25,26^. These methods adopted previously may fail to recognize the correct RLS due to experimental errors or insufficient precision. For the Tafel analysis, it is hard to tell the RLS in CO_2_ER filed due to mass transport limitations^16^ and the value assumed for the transfer coefficient (α)^27^. Because the theoretical Tafel slope might be incorrect due to the improper assumption of 0.5 transfer coefficient which might lead to a wrong guidance for the RLS^27^. The reaction order analysis used to determine whether the RLS includes a proton transfer step is often performed by changing the pH of the electrolyte^28,29^. It is an incomplete approach because the source of protons may come from water molecules instead of H^+^^[ 30^, where the activity of CO_2_ER would not change with pH. On the other hand, the RLS without proton transfer may still change with pH if the proton transfer step occurs before the RLS. Although theoretical calculations have widely been adopted to find the RLS, the reliability of the results greatly depends on the choice of models and experimental methods^31,32^. Therefore, it is of great significance to also develop a reliable experimental strategy to reveal the RLS for CO_2_ER.

In this work, theoretical derivations and experimental results are combined and discussed in detail to provide sufficient evidence for the determination of the RLS during the two-electron transfer CO_2_ER. The rate expressions with different RLSs were first derived through the breakdown of the Butler-Volmer equation. By analyzing these rate expressions, an effective method for discovering the RLS was proposed, which combines pH dependency and kinetic isotope effect (KIE) experiments. Since H^+^ ions and H_2_O molecules are both proton donors, the reaction orders of H^+^ and H_2_O can be obtained by changing the pH of the electrolyte and the adoption of D_2_O in the electrolyte. Corresponding results could help clarify whether the adsorption of CO_2_ with its concomitant electron transfer (ET) step or the other possible steps, such as the proton transfer (PT), proton-coupled electron transfer (PCET), or desorption (D) of product, is the RLS. Subsequently, Au, Ag, Sn, and In were used as model catalysts to reveal the RLS of the two-electron transfer CO_2_ER. For all the electrocatalysts, the current densities of CO (*j*_CO_) and formate (*j*_HCOO−_) are independent of both pH and deuteration of the electrolyte, which indicates the CO_2_ adsorption step to be the RLS.

## Results

### The rate expressions with different reaction steps as the RLS

In order to discover the RLS, the Butler-Volmer equation was employed to describe the kinetic rate expression of two-electron transfer CO_2_ER. It describes how the electrical current passing through an electrode depends on the voltage difference between the electrode and the bulk electrolyte for simple unimolecular redox reactions, when both a cathodic and an anodic reaction proceeding on the same electrode are controlled by surface reactions rather than the mass transfer of electrolyte^33^. For electroreduction reactions (Eq. 1, where O_*x*_ and R_ed_ represent oxidant and reductant, respectively), the Butler-Volmer equation is shown as Eq. 2^34^.

1 $${{{{{\rm{O}}}}}}_{{{{{\rm{x}}}}}}+{{ne}}^{-}\to {{{{{\rm{R}}}}}}_{{{{{\rm{ed}}}}}}$$

2 $$j=nF{k}_{b}^{0}a[{{{{{{\rm{R}}}}}}}_{{{{{{\rm{ed}}}}}}}]\exp [(1 - \alpha )f\eta ] - {{{{{\rm{n}}}}}}F{k}_{f}^{0}a[{{{{{{\rm{O}}}}}}}_{{{{{{\rm{x}}}}}}}]\exp [ - \alpha f\eta ]$$

In Eq. 2, *j* is the current density; *η* is the overpotential for the cathodic reaction; *k*_*f*_^*0*^ is the standard forward rate constant; *k*_*b*_^*0*^ is the standard backward rate constant; *F* is the Faraday constant; *f = F/RT*, where *R* is the ideal gas constant and *T* is absolute temperature; *α* is the transfer coefficient assumed to be equal to 0.5; *n* is the number of transferred electrons; *a*[R_ed_] and *a*[O_x_] are the concentrations of reductant and oxidant.

When the overpotential is sufficiently high, i.e., exp[–(1–*α*)*f*$\eta$] << exp(–*αf*$\eta$), the backward reaction can be ignored^35^. Even the high-performance CO_2_ electrolysis catalysts have sufficient overpotentials to meet this condition^36^. Therefore, Eq. 2 can be simplified to Eq. 3. At equilibrium conditions (*j* = 0), Eq. 2 can be simplified to Eq. 4.

3 $$j= - nF{k}_{f}^{0}a[{{{{{{\rm{O}}}}}}}_{{{{{{\rm{x}}}}}}}]\exp ( - \alpha f\eta )$$

4 $$a[{{{{{{\rm{R}}}}}}}_{{{{{{\rm{ed}}}}}}}]/a[{{{{{{\rm{O}}}}}}}_{{{{{{\rm{x}}}}}}}]={K}^{\theta }\exp ( - f\eta )$$

By combining Eq. 4, the *a*[O_x_] in Eq. 3 can be represented by the concentration of reactants and *K*^*θ*^exp(–*f*$\eta$) in the previous step (see the supplementary information for more details). Subsequently, the rate expression of the two-electron transfer CO_2_ER with a specific reaction step as the RLS can be derived (Tables 1 and 2, different labels are assigned to the corresponding RLSs according to the reaction processes). Whether the RLS is controlled by ET, PT, PCET, or D is also shown in the Tables. One thing should also be kept in mind is that all these expressions are based on assumptions of what might happen in the mechanism, which may not cover all possible kinetic cases at current cognitive levels.

**Table 1:** Reaction kinetic parameters for different possible RLSs during the two-electron transfer CO_2_ER to CO^a^.

Step^b^	Possible RLS	Proposed Rate Expression^c^	Type^d^	H^+^ order	H_2_O order	Tafel slope^e^ (mV/dec)
A1	CO_2_ + * + e^−^ $\to$ *CO_2_^−^	*j*_CO_ *=* 2*Fk*_*A1*_^*0*^*a*[CO_2_]θ^*^exp(*−αfη*)	ET	0	0	118
A2	*CO_2_^−^ + H^+^ $\to$ *COOH	*j*_CO_ *=* 2*Fk*_*A2*_^*0*^*K*_*A1*_^*θ*^*a*[CO_2_]θ^*^a[H^+^]exp(*−fη*)	PT	1	0	59
A3	*COOH + e^−^ $\to$ *COOH^−^	*j*_CO_ *=* 2*Fk*_*A3*_^*0*^*K*_*A2*_^*θ*^*K*_*A1*_^*θ*^*a*[CO_2_]θ^*^a[H^+^]exp[−(1*+α*)*fη*]	ET	1	0	39
A4	*COOH^−^ + H^+^ $\to$ *CO + H_2_O	*j*_CO_ *=* 2*Fk*_*A4*_^*0*^*K*_*A3*_^*θ*^*K*_*A2*_^*θ*^*K*_*A1*_^*θ*^*a*[CO_2_]θ^*^a^2^[H^+^]exp(−2*fη*)	PT	2	0	30
A5	*CO $\to$ CO + *	*j*_CO_ *=* 2*Fk*_*A4*_^*0*^*K*_*A3*_^*θ*^*K*_*A2*_^*θ*^*K*_*A1*_^*θ*^*a*[CO_2_]θ^*^a^2^[H^+^]exp(−2*fη*)/*a*(H_2_O)	D	2	−1	30
a1	CO_2_ + * + e^−^ $\to$ *CO_2_^−^	*j*_CO_ = 2*Fk*_*a1*_^*0*^*a*[CO_2_]θ^*^exp(*−αfη*)	ET	0	0	118
a2	*CO_2_^−^ + H_2_O $\to$ *COOH + OH^−^	*j*_CO_ *=* 2*Fk*_*a2*_^*0*^*K*_*a1*_^*θ*^*a*[CO_2_]θ^*^a[H_2_O]exp(*−fη*)	PT	0	1	59
a3	*COOH + e^−^ $\to$ *COOH^−^	*j*_CO_ *=* 2*Fk*_*a3*_^*0*^*K*_*a2*_^*θ*^*K*_*a1*_^*θ*^*a*[CO_2_]θ^*^a[H^+^]*a*[H_2_O]exp[−(1+*α*)*fη*]/*K*_*W*_	ET	1	1	39
a4	*COOH^−^ $\to$ *CO + OH^−^	*j*_CO_ *=* 2*Fk*_*a4*_^*0*^*K*_*a3*_^*θ*^*K*_*a2*_^*θ*^*K*_*a1*_^*θ*^*a*[CO_2_]θ^*^a[H^+^]*a*[H_2_O]exp(−2*fE*)/*K*_*W*_	PT	1	1	30
a5	*CO $\to$ CO + *	*j*_CO_ *=* 2*Fk*_*a5*_^*0*^*K*_*a4*_^*θ*^*K*_*a3*_^*θ*^*K*_*a2*_^*θ*^*K*_*a1*_^*θ*^*a*[CO_2_]θ^*^a^2^[H^+^]*a*[H_2_]exp(−2*fη*)/*K*_*W*_^2^	D	2	1	30
B1	CO_2_ + * + e^−^ + H^+^ $\to$ *COOH	*j*_CO_ *=* 2*Fk*_*B1*_^*0*^*a*[CO_2_]θ^*^a[H^+^]exp(−*αfE*)	PCET	1	0	118
B2	*COOH + e^−^ $\to$ *COOH^−^	*j*_CO_ *=* 2*Fk*_*B2*_^*0*^*K*_*B1*_^*θ*^*a*[CO_2_]θ^*^a[H^+^]exp[−(1+*α*)*fη*]	ET	1	0	39
B3	*COOH^−^ + H^+^ $\to$ *CO + H_2_O	*j*_CO_ *=* 2*Fk*_*B3*_^*0*^*K*_*B2*_^*θ*^*K*_*B1*_^*θ*^*a*[CO_2_]θ^*^a^2^[H^+^]exp(−2*fE*)	PT	2	0	30
B4	*CO $\to$ CO + *	*j*_CO_ *=* 2*Fk*_*B4*_^*0*^*K*_*B3*_^*θ*^*K*_*B2*_^*θ*^*K*_*B1*_^*θ*^*a*[CO_2_]θ^*^a^2^[H^+^]exp(−2*fη*)/*a*[*H*_*2*_*O*]	D	2	−1	30
b1	CO_2_ + * + e^−^ + H_2_O $\to$ *COOH + OH^−^	*j*_CO_ *=* 2*Fk*_*b1*_^*0*^*a*[CO_2_]θ^*^a[H_2_O]exp(*−αfη*)	PCET	0	1	118
b2	*COOH + e^−^ $\to$ *COOH^−^	*j*_CO_ *=* 2*Fk*_*b2*_^*0*^*K*_*b1*_^*θ*^*a*[CO_2_]θ^*^a[H_2_O]*a*[H^+^]exp[−(1+*α*)*fη*]/*K*_*W*_	ET	1	1	39
b3	*COOH^−^ $\to$ *CO + OH^−^	*j*_CO_ *=* 2*Fk*_*b3*_^*0*^*K*_*b2*_^*θ*^*K*_*b1*_^*θ*^*a*[CO_2_]θ^*^a[H_2_O]*a*[H^+^]exp(−2*fη*)/*K*_*W*_	PT	1	1	30
b4	*CO $\to$ CO + *	*j*_CO_ *=* 2*Fk*_*b4*_^*0*^*K*_*b3*_^*θ*^*K*_*b2*_^*θ*^*K*_*b1*_^*θ*^*a*[CO_2_]θ^*^a[H_2_O]*a*^2^[H^+^]exp(−2*fη*)/*K*_*W*_^2^	D	2	1	30
C1	CO_2_ + * + e^−^ + H^+^ $\to$ *COOH	*j*_CO_ *=* 2*Fk*_*C1*_^*0*^*a*[CO_2_]θ^*^a[H^+^]exp(−*αfη*)	PCET	1	0	118
C2	*COOH + e^−^ + H^+^ $\to$ *CO + H_2_O	*j*_CO_ *=* 2*Fk*_*C2*_^*0*^*K*_*C1*_^*θ*^*a*[CO_2_]θ^*^a^2^[H^+^]exp[−(1+*α*)*fη*]	PCET	2	0	39
C3	*CO $\to$ CO + *	*j*_CO_ *=* 2*Fk*_*C3*_^*0*^*K*_*C2*_^*θ*^*K*_*C1*_^*θ*^*a*[CO_2_]θ^*^a^2^[H^+^]exp(−2*fη*)/*a*(H_2_O)	D	2	−1	30
c1	CO_2_ + * + e^−^ + H_2_O $\to$ *COOH + OH^−−^	*j*_CO_ *=* 2*Fk*_*c1*_^*0*^*a*[CO_2_]θ^*^a[H_2_O]exp(*−αfη*)	PCET	0	1	118
c2	*COOH + e^−^ $\to$ *CO + OH^−^	*j*_CO_ *=* 2*Fk*_*c2*_^*0*^*K*_*c1*_^*θ*^*a*[CO_2_]θ^*^a[H_2_O]*a*[H^+^]exp[−(1+*α*)*fη*]/*K*_*W*_	PCET	1	1	39
c3	*CO $\to$ CO + *	*j*_CO_ *=* 2*Fk*_*c3*_^*0*^*K*_*c2*_^*θ*^*K*_*c1*_^*θ*^*a*[CO_2_]θ^*^a[H_2_O]*a*^2^[H^+^]exp(−2*fη*)/*K*_*W*_^2^	D	2	1	30

^a^CO_2_ + 2H^+^ + 2e^−^ $\to$ CO + H_2_O or CO_2_ + 2H_2_O + 2e^−^ $\to$ CO + 2OH^−^.

^b^Capital letters indicate that the source of protons is H^+^, lower case letters indicate that the source of protons is H_2_O.

^c^*k*^*0*^ is the standard forward rate constant*; f = F/RT; η* is the overpotential for the cathodic reaction; *α* is the transfer coefficient; *F* is Faraday constant and equals to 96,485 C/mol; *n* is the number of reactive transfer electrons; *R* is the ideal gas constant and equals to 8.314 J/(mol K); *T* is the absolute temperature.

^d^*ET* electron transfer, *PT* proton transfer, *PCET* proton-coupled electron transfer, *D* desorption

^e^Assuming *α* = 0.5.

**Table 2:** Reaction kinetic parameters for different possible RLSs during the two-electron transfer CO_2_ER to formate^a^.

Step^b^	Possible RLS	Proposed rate expression^c^	Type^d^	H^+^ order	H_2_O order	Tafel slope^e^ (mV/dec)
D1	CO_2_ + * + e^−^ $\to$ *CO_2_^−^	*j*_HCOO_^−^ *=* 2*Fk*_*D1*_^*0*^*a*[CO_2_]θ^*^exp(−*αfη*)	ET	0	0	118
D2	*CO_2_^−^ + H^+^ $\to$ *OOCH	*j*_HCOO_^−^ *=* 2*Fk*_*D2*_^*0*^*K*_*D1*_^*θ*^*a*[CO_2_]θ^*^a[H^+^]exp(−*fη*)	PT	1	0	59
D3	*OOCH + e^−^ $\to$ *OOCH^−^	*j*_HCOO_− *=* 2*Fk*_*D3*_^*0*^*K*_*D2*_^*θ*^*K*_*D1*_^*θ*^*a*[CO_2_]θ^*^a[H^+^]exp[−(1+*α*)*fη*]	ET	1	0	39
D4	*OOCH^−^ $\to$ HCOO^−^ + *	*j*_HCOO_−*=* 2*Fk*_*D4*_^*0*^*K*_*D3*_^*θ*^*K*_*D2*_^*θ*^*K*_*D1*_^*θ*^*a*[CO_2_]θ^*^a[H^+^]exp(−2*fη*)	PT	1	0	30
d1	CO_2_ + * + e^−^ $\to$ *CO_2_^−^	*j*_HCOO_− = 2*Fk*_*d1*_^*0*^*a*[CO_2_]θ^*^exp(*−αfη*)	ET	0	0	118
d2	*CO_2_− + H_2_O $\to$ *OOCH + OH^−^	*j*_HCOO_− = 2*Fk*_*d2*_^*0*^*K*_*d1*_^*θ*^*a*[CO_2_]θ^*^a[H_2_O]exp(−f*η*)	PT	0	1	59
d3	*OOCH + e^−^ $\to$ *OCOH^−^	*j*_HCOO_− = 2*Fk*_*d3*_^*0*^*K*_*d2*_^*θ*^*K*_*d1*_^*θ*^*a*[CO_2_]θ^*^a[H^+^]*a*[H_2_O]exp[−(1+α)*fη*]/*K*_*W*_	ET	1	1	39
d4	*OOCH^−^ $\to$ HCOO^−^ + *	*j*_HCOO_− = 2*Fk*_*d4*_^*0*^*K*_*d3*_^*θ*^*K*_*d2*_^*θ*^*K*_*d1*_^*θ*^*a*[CO_2_]θ^*^a[H^+^]*a*[H_2_O]exp[−2*fη*]/*K*_*W*_	PT	1	1	30
E1	CO_2_ + * + e^−^ + H^+^ $\to \,$*OOCH	*j*_HCOO_− *=* 2*Fk*_*E1*_^*0*^*a*[CO_2_]θ^*^a[H^+^]exp(−*αfη*)	PCET	1	0	118
E2	*OOCH + e^−^ $\to$ *OOCH^−^	*j*_HCOO_− *=* 2*Fk*_*E2*_^*0*^*K*_*E1*_^*θ*^*a*[CO_2_]θ^*^a[H^+^]exp[−(1+*α*)*fη*]	ET	1	0	39
E3	*OOCH^−^ $\to$ HCOO^−^ + *	*j*_HCOO_− *=* 2*Fk*_*E3*_^*0*^*K*_*E2*_^*θ*^*K*_*E1*_^*θ*^*a*[CO_2_]θ^*^a[H^+^]exp[−2*fη*]	PT	1	0	30
e1	CO_2_ + * + e^−^ + H_2_O $\to$ *OOCH + OH^−^	*j*_HCOO_− *=* 2*Fk*_*e1*_^*0*^*a*[CO_2_]θ^*^a[H_2_O]exp(−*αfη*)	PCET	0	1	118
e2	*OOCH + e^−^ $\to$ *OOCH^−^	*j*_HCOO_− *=* 2*Fk*_*e2*_^*0*^*K*_*e1*_^*θ*^*a*[CO_2_]θ^*^a[H_2_O]*a*[H^+^]exp[−(1+*α*)*fη*]/*K*_*W*_	ET	1	1	39
e3	*OOCH^−^ $\to$ HCOO^−^ + *	*j*_HCOO_− *=* 2*Fk*_*e3*_^*0*^*K*_*e2*_^*θ*^*K*_*e1*_^*θ*^*a*[CO_2_]θ^*^a[H_2_O]*a*[H^+^]exp(−2*fη*)/*K*_*W*_	PT	1	1	30

^a^CO_2_ + H^+^ + 2e^−^ $\to$ HCOO^−^ or CO_2_ + H_2_O + 2e^−^ $\to$ HCOO^−^ + OH^−^

^b^Capital letters indicate that the source of protons is H^+^, lower case letters indicate that the source of protons is H_2_O.

^c^*k*^*0*^ is the standard forward rate constant*; f = F/RT; η* is the overpotential for the cathodic reaction; *α* is the transfer coefficient; *F* is Faraday constant and equals to 96,485 C/mol; *n* is the number of reactive transfer electrons; *R* is the ideal gas constant and equals to 8.314 J/(mol K); *T* is the absolute temperature.

^d^*ET* electron transfer, *PT* proton transfer, *PCET* proton-coupled electron transfer, *D* desorption.

^e^Assuming *α* = 0.5.

According to the rate expression, the reaction order of different reactants can be obtained. For example, when the adsorption of CO_2_ with the ET (step A1 in Table 1, Eq. 5) is the RLS for CO_2_ER to CO,

5 $${{{{{{\rm{CO}}}}}}}_{2}+\ast +{e}^{-}\to \ast {{{{{\rm{CO}}}}}}_{2}^{-}$$

the rate expression (Eq. 6) is

6 $${j}_{{{{{\rm{co}}}}}}=2F{k}_{A1}^{0}a[{{{{{{\rm{CO}}}}}}}_{2}]{\theta }^{\ast }\exp ( - \alpha f\eta ),$$

where the corresponding reaction order of H^+^ and H_2_O molecules should be 0.

Thus, the RLS of two-electron transfer CO_2_ER could be determined via the analysis of the reaction order of the reactants^19,28,30,37^. Whether the reaction is controlled by the concentration of H^+^ can be reflected by its pH dependency. However, whether protons are involved in the RLS cannot be simply determined by the pH dependency of the reaction, since H_2_O could be the proton source. Therefore, KIE experiments can be conducted to reveal if the H_2_O molecules are involved in the reaction as a proton source. It is noteworthy that possible RLS with the rate expression involving neither H^+^ nor H_2_O may still be controlled by them because protons may take part in the reaction processes before the RLS (see the supplementary information for more details). Therefore, in-depth reaction rate analysis is essential for the determination of the RLS.

### The fabrication of model catalysts

To elucidate the RLS of two-electron transfer CO_2_ER, Au, Ag, Sn, and In were chosen as model catalysts. Au and Ag have been proved to exhibit good performance for the production of CO, and In and Sn are promising catalysts with high selectivity for formate^38,39^. These catalysts were deposited on Si(100) wafers by magnetron sputtering. To enhance the adhesion between the catalysts and the Si wafers, Ti films with a thickness of approximately 15 nm were first deposited on the Si wafers^40^. Catalysts with relatively high conductivity (i.e., Au and Ag) were directly deposited on the Ti films. The thicknesses of the catalyst films were controlled to be 200 nm. Less conductive In and Sn catalyst films with a thickness of about 400 nm were deposited after the adhesion of 30 nm Au layers onto the Ti films. This strategy improves the conductivity of the substrate and preventes delamination of the films under cathodic potentials. Because of the high surface tension of Sn and In, thicker films need to be deposited to cover the substrates completely. According to scanning electron microscopy (SEM) images (Supplementary Fig. 1), the Au, Ag, In, and Sn catalyst films are evenly distributed over the substrates. X-ray diffraction (XRD) patterns (Supplementary Fig. 2) show that these films have polycrystalline structures. No signal of the substrate materials was found in the survey X-ray photoelectron spectroscopy (XPS) spectra of the samples, indicating that no substrate would be exposed to the electrolyte (Supplementary Fig. 3). Furthermore, the analyses of the surface valence states of the Au and Ag films show that they are primarily in the metallic state with only slight surface oxidation for Ag. However, the surfaces of the Sn and In films were oxidized (Supplementary Fig. 4). These four model catalysts were then used to study the RLS of two-electron transfer CO_2_ER. Here, CO and H_2_ were the main products over Ag and Au. CO, formate, and H_2_ were the main products over Sn and In. The total Faradaic efficiency is basically equal to 100% (Supplementary Fig. 5).

### Experimental determination of the RLS

**Fig. 1:**
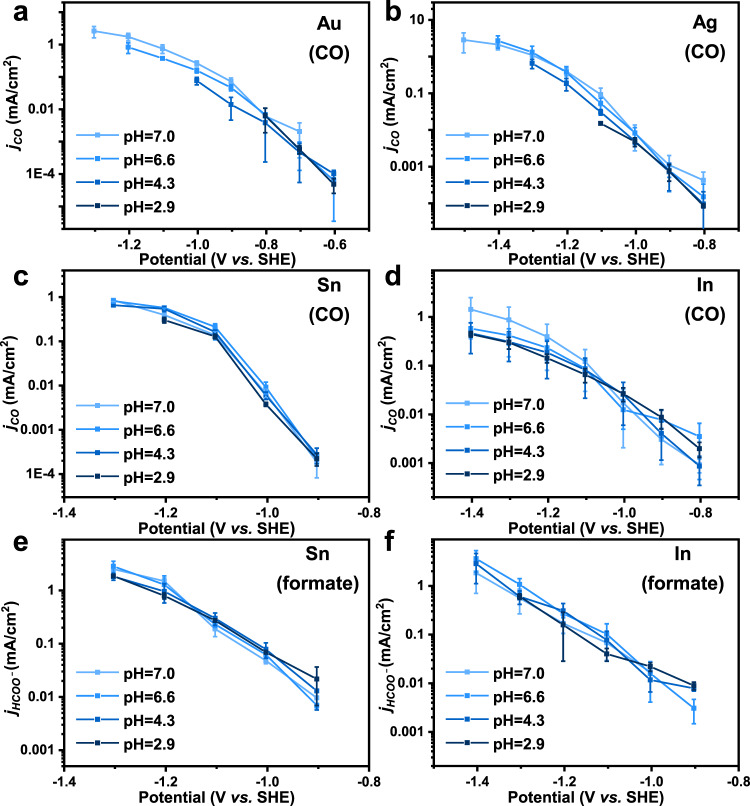
The pH dependency for CO_2_ER toward CO and formate. The *j*_CO_ for Au (**a**), Ag (**b**), Sn (**c**), and In (**d**) catalysts in electrolytes with different pH. The *j*_HCOO_− for Sn (**e**) and In (**f**) catalysts in electrolytes with different pH. Error bars are means ± standard deviation (*n* = 3 replicates).

In order to explore the impact of the H^+^ concentration on the two-electron transfer CO_2_ER, the changing trends of *j*_CO_ and *j*_HCOO−_ with the variation of electrolyte pH were analyzed. Experiments were carried out in a flowing H-cell with five types of CO_2_ saturated electrolytes (0.3 M KHCO_3_, pH 7.0; 0.1 M K_3_PO_4_, pH 6.6; 0.1 M KH_2_PO_4_, pH 4.3; 0.1 M KH_2_PO_4_ + 0.1 M H_3_PO_4_, pH 2.9; 0.1 M H_3_PO_4_, pH 1.6). Figure 1a–d show the *j*_CO_ of the four catalysts as a function of applied potential. The electrocatalytic activity for CO generation is barely affected by the pH of the electrolytes (Supplementary Fig. 6a). Since the *j*_CO_ is consistent under various pH from 2.9 to 7.0, the reaction rate expression of CO_2_ER to CO should not include H^+^. Therefore, only the following reaction steps (Eqs. 7–9), with reaction order for H^+^ to be 0 in the rate expressions, could possibly be the RLS.

7 $${{{{{\rm{A}}}}}}1\,{{{{{\rm{or}}}}}}\,{{{{{\rm{a}}}}}}1:{{{{{{\rm{CO}}}}}}}_{2}+\ast +{{{{{{\rm{e}}}}}}}^{-}\to \ast {{{{{{{\rm{CO}}}}}}}_{2}}^{-}$$

8 $${{{{{\rm{a}}}}}}2:\ast {{{{{{{\rm{CO}}}}}}}_{2}}^{-}+{{{{{{\rm{H}}}}}}}_{2}{{{{{\rm{O}}}}}}\to \ast {{{{{\rm{COOH}}}}}}+{{{{{{\rm{OH}}}}}}}^{-}$$

9 $${{{{{\rm{b}}}}}}1\,{{{{{\rm{or}}}}}}\,{{{{{\rm{c}}}}}}1:{{{{{{\rm{CO}}}}}}}_{2}+\ast +{e}^{-}+{{{{{{\rm{H}}}}}}}_{2}{{{{{\rm{O}}}}}}\to \ast {{{{{\rm{COOH}}}}}}+{{{{{{\rm{OH}}}}}}}^{-}$$

Similar to the case of CO, the *j*_HCOO_^−^ of both Sn and In did not show significant change with the electrolyte pH (Fig. 1e, f and Supplementary Fig. 6b). Thus, the reaction order of H^+^ for CO_2_ER to formate should also be 0. According to Table 2, the possible RLS meeting this requirement are listed as follows (Eqs. 10–12).

10 $${{{{{\rm{D}}}}}}1\,{{{{{\rm{or}}}}}}\,{{{{{\rm{d}}}}}}1:{{{{{{\rm{CO}}}}}}}_{2}+\ast +{{{{{{\rm{e}}}}}}}^{-}\to \ast {{{{{{{\rm{CO}}}}}}}_{2}}^{-}$$

11 $${{{{{\rm{d}}}}}}2:\ast {{{{{{{\rm{CO}}}}}}}_{2}}^{-}+{{{{{{\rm{H}}}}}}}_{2}{{{{{\rm{O}}}}}}\to \ast {{{{{\rm{OOCH}}}}}}+{{{{{{\rm{OH}}}}}}}^{ - }$$

12 $${{{{{\rm{e}}}}}}1:{{{{{{\rm{CO}}}}}}}_{2}+\ast +{{{{{{\rm{e}}}}}}}^{ - }+{{{{{{\rm{H}}}}}}}_{2}{{{{{\rm{O}}}}}}\to \ast {{{{{\rm{OOCH}}}}}}+{{{{{{\rm{OH}}}}}}}^{ - }$$

**Fig. 2:**
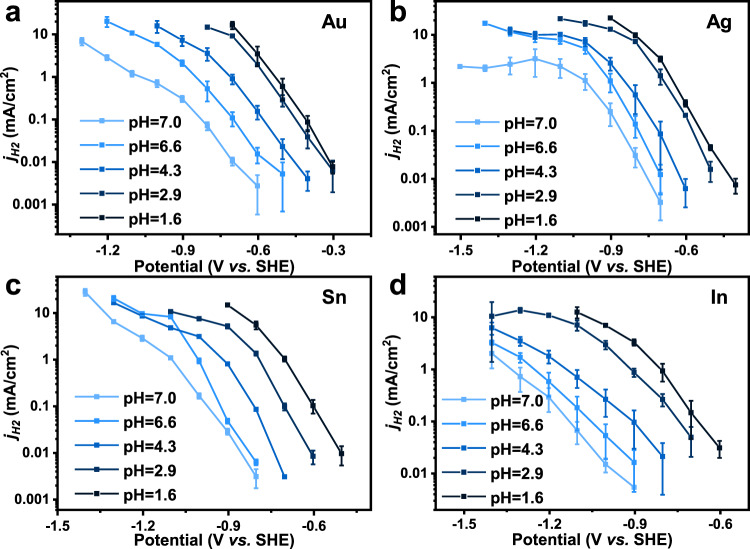
The pH dependency for HER. The *j*_H2_ for Au (**a**), Ag (**b**), Sn (**c**), and In (**d**) catalysts in electrolytes with different pH. Error bars are means ± standard deviation (*n* = 3 replicates).

On the contrary, the activity of the hydrogen evolution reaction (HER) is enhanced as the pH decreases (Fig. 2 and Supplementary Fig. 7). This phenomenon indicates that the RLS of HER depends on the concentration of H^+^, which is consistent with the results from the literatures^30,41^. This result further supports the feasibility of revealing the RLS of two-electron transfer CO_2_ER by pH dependency.

Considering the inevitable deviation of the bulk pH and local pH caused by the constant consumption of protons during CO_2_ER and HER, the local pH was investigated. Supplementary Fig. 9 shows the local pH as a function of the operating potential for In (see the note of Supplementary Fig. 9 for the reason why choosing In as the representative catalyst). The local pH elevates slowly with the increase of the reaction potential in different electrolytes. However, the order of the local pH is the same as that of the bulk pH. Therefore, it is reasonable to use bulk pH in the current work. Also, regarding the inevitable change of ion concentration when changing pH, comparative experiments in 0.1 and 0.3 M KH_2_PO_4_ show the *j*_CO_ and *j*_HCOO_^−^ are basically the same (Supplementary Fig. 10), indicating the change in the concentrations of potassium and phosphate has little effect on the CO_2_ER activity in those experiments. So, the ion concentration caused by changing the pH will not affect the conclusion.

The pH dependency experiments eliminated many potential RLSs. To further determine the RLS, KIE experiments were considered to analyze whether water molecules are involved in the rate expression. Since the KIE of CO_2_ER in homogeneous catalysts was tested to be 6.92^42^ and 8.2^43^ for CO and formate, respectively, which means if proton was involved in the RLS for CO_2_ER to CO or formate, the KIE should be >1. Secondly, the KIE was used to exclude the step of H_2_O providing proton, where the KIE of H_2_O dissociation are around 3.4–7.6^44^, so the involvement of protons in the RLS of CO_2_ER should show the KIE>1. Thirdly, KIE experiments have been known to lead to false-negative conclusions, but only in very specific and rare instancesnormally involving more environment-sensitive molecular catalysts^45^. To the best of our knowledge, there have been no calculation on transition-metal catalysts showing D_2_O could distort CO_2_ electrolysis results for two-electron products compared to using H_2_O. Therefore, it seems reasonable to use KIE to explore whether water molecules are involved in the rate expression.

**Fig. 3:**
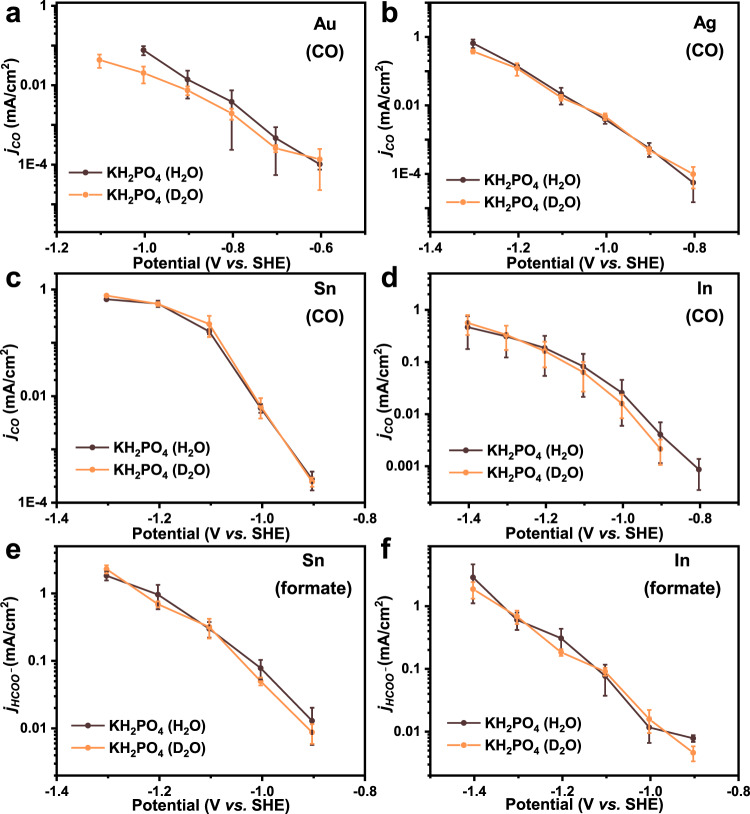
Kinetic isotope effect for CO_2_ER toward CO and formate. The *j*_CO_ for Au (**a**), Ag (**b**), Sn (**c**), and In (**d**) in 0.1 M KH_2_PO_4_ electrolytes with D_2_O and H_2_O. The *j*_HCOO_^−^ for Sn (**e**) and In (**f**) in 0.1 M KH_2_PO_4_ electrolytes with D_2_O and H_2_O. Error bars are means ± standard deviation (*n* = 3 replicates).

**Fig. 4:**
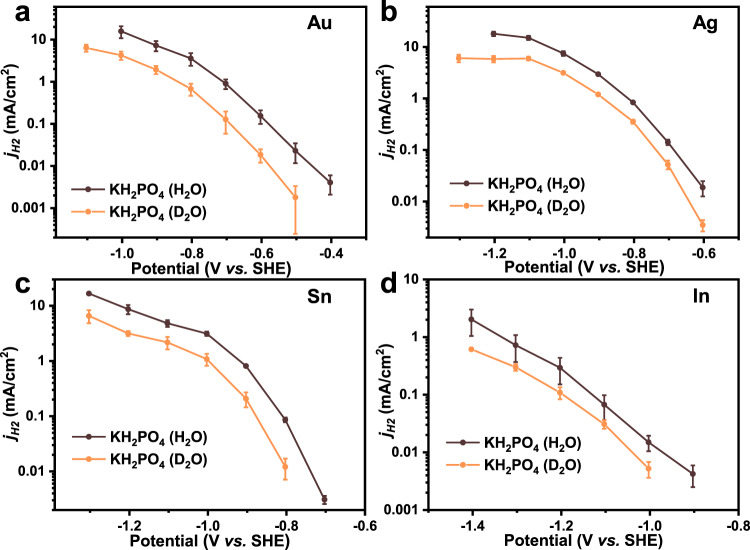
Kinetic isotope effect for HER. The *j*_H2_ for Au (**a**), Ag (**b**), Sn (**c**) and In (**d**). The data were obtained from running CO_2_ER experiments at 0.1 M KH_2_PO_4_ electrolyte with D_2_O and H_2_O. Error bars are means ± standard deviation (*n* = 3 replicates).

In the KIE experiments, both *j*_CO_ and *j*_HCOO_^−^ did not change with the use of D_2_O instead of H_2_O in the 0.1 M KH_2_PO_4_ electrolyte for Au, Ag, Sn, and In catalysts (Fig. 3). To eliminate the possibility that KH_2_PO_4_ provides protons that equilibrate with D_2_O to generate a small amount of H_2_O, K_2_CO_3_ solutions in H_2_O and D_2_O were also chosen as electrolytes. The results of activity tests with the In catalyst show that *j*_CO_ and *j*_HCOO_^−^ are almost the same in both electrolytes (Supplementary Fig. 11a,b). These phenomena demonstrate that the reaction order of H_2_O for CO_2_ER to CO or formate should be 0. Combining this knowledge with the results of pH dependency studies, the RLS of both the two-electron transfer CO_2_ER to CO and formate is deduced to be the adsorption of CO_2_ with one electron transferred simultaneously, as shown in Eqs. 7 and 10, respectively. KIE experiments for HER were also conducted with the Au, Ag, Sn, and In catalysts. When D_2_O was used in the solvent, the *j*_H2_ drops significantly (Fig. 4 and Supplementary Fig. 11c), indicating water molecules are a part of the RLS or take part in the reaction process before the RLS, which is also consistent with previous reports^46^. It should be noticed that the pH dependency and KIE experiments were not conducted in alkaline electrolytes since the solution would become neutral or acidic after the dissolution of CO_2_^47^, but we think the conclusion might also be applicable to alkaline solutions (see Supplementary Figs. 12 and 13 for detailed explanation).

Based on the above analyses, the RLS of two-electron transfer CO_2_ER for Au, Ag, Sn, and In catalysts was found to be the adsorption of CO_2_ with the concomitant ET. Since the transfer of electrons is quite fast over metal catalysts^28,48^, the ultimate RLS is proposed to be most likely the adsorption of CO_2_.

## Discussion

In summary, this work presents an effective approach to determine the RLS by employing a detailed analysis of the reaction rate expression together with pH dependency and KIE experiments. It was found that both *j*_CO_ and *j*_HCOO_^−^ are independent of the pH and deuteration of the electrolytes for Au, Ag, Sn, and In, which are representative catalysts for two-electron transfer CO_2_ER. The results reveal that the RLS of the two-electron CO_2_ER should be the adsorption of CO_2_. This finding suggests effective strategies to design highly active CO_2_ER catalyst for the production of CO and formate.

## Methods

### Electrode preparation

Au, Ag, In, and Sn thin films were deposited onto single-crystal Si wafers with the (100) orientation using an AJA ATC Orion-5 magnetron sputtering system. Before the deposition, the Si wafers were etched with Ar^+^ ions for 5 min with a power of 40 W to clean the silicon oxide on Si wafers. Then, 15 nm Ti films were deposited as binders between catalysts and Si wafers at the power of 130 W. Au and Ag catalyst films with the thickness of about 200 nm were deposited over Ti at 50 W. Due to the poor electrical conductivity of In and Sn catalysts, Au films (30 nm) were added over Ti before depositing 400 nm of the catalyst films at 20 W.

### Electrode characterization

The crystal structures of the Au, Ag, In, and Sn thin films were analyzed with a Rigaku Smartlab X-ray diffractometer (XRD) using Cu Kα radiation (40 kV, 40 mA). The near-surface compositions of the thin films were measured with a Kratos Axis Ultra DLD X-ray photoelectron spectrometer (XPS). All spectra were acquired using monochromatized Al Kα radiation (15 kV, 15 mA). The kinetic energy scale of the measured spectra was calibrated by setting the C 1s binding energy to 284.8 eV. The surface structure of those thin films was recorded using an FEI XL30 Sirion scanning electron microscope (SEM) at the 5 kV acceleration voltage, Everhart-Thornley detector, secondary electrons mode.

### Electrochemical characterization

All electrochemical activity measurements were conducted in a custom electrochemical cell machined from PEEK at room temperature and atmospheric pressure. The cell was sonicated in 20 wt% nitric acid and thoroughly rinsed with DI water before all experimentation. The working and counter electrodes were parallel and separated by a bipolar membrane (Fumasep FBM). The exposed geometric surface area of each electrode was 1 cm^2^. The electrolyte volumes in the cathodic and anodic chambers were 6 mL and 1 mL, respectively. The counter electrode was iridium dioxide (IrO_2_) purchased from Dioxide Materials. The working electrode potential was referenced against a miniature Ag/AgCl electrode (Innovative Instruments Inc.) that was calibrated against a homemade standard hydrogen electrode (SHE). 0.3 M potassium bicarbonate (KHCO_3_, Sigma Aldrich 99.7%, pH 7.0), 0.1 M potassium phosphate (K_3_PO_4_, Sigma Aldrich 99.99%, pH 6.6), 0.1 M monopotassium phosphate (KH_2_PO_4_, Sigma Aldrich 99.99%, pH 4.3), 0.1 M monopotassium phosphate adjusted the pH with 0.1 phosphoric acid (KH_2_PO_3_ with H_3_PO_4_, pH 2.9) and 0.1 M phosphoric acid (H_3_PO_4_, Sigma Aldrich 85% w/w, pH 1.6) solutions prepared using 18.2 MΩ·cm Milli-Q water were used as the cathodic electrolyte. 0.1 M KH_2_PO_4_ was used as the anodic electrolyte. Metallic impurities in the as-prepared electrolytes were removed before electrolysis by chelating them with Chelex 100 (Sigma Aldrich). The cathodic electrolyte was sparged with CO_2_ (99.999% Praxair Inc.) at a rate of 10 sccm for 30 min prior to the experiments. Then CO_2_ was pumped into the cathodic chamber by using a peristaltic pump (SHENCHEN LabN6) with the rate of 130 rev/min. Here, the experiments process in this pump speed cannot be the significantly diffusion-limited, since we have obtained relatively straight Tafel slopes over three orders of magnitude in current (Figs. 1–4). The pH values of electrolytes were measured in CO_2_ saturated solutions.

The produced CO and H_2_ were tested by gas chromatography (GC, Thermo scientific, TRACE 1300). Ar was used as the carrier gas. The GC was equipped with a packed Molsieve 5A column, a packed Hayesep Q column, and a Rt-Qbond column to separate the gaseous products. Thus, H_2_ and CO could be identified using a thermal conductivity detector and a flame ionization detector, respectively. The liquid-phase products were analyzed after the electrolysis using a high-performance liquid chromatography (HPLC, Agilent 1200 series). Liquid-phase products were separated by an Aminex HPX-87H column (Bio-Rad) that was maintained at 50 °C. The HPLC was equipped with a diode array detector (DAD) and a refractive index detector (RID). The response signals of the DAD and RID were calibrated by solutions with different concentrations.

Electrochemical characterizations were performed using a Biologic VSP-300 potentiostat. All electrochemical measurements were recorded versus the reference electrode and converted to the SHE scale. Current interrupt was used to determine the uncompensated resistance (*R*_u_) of the electrochemical cell. The accurate potentials were corrected according to the *R*_u_ (see Supplementary Table 3 for detailed potential correction process).

The electrocatalytic activity of the thin films was assessed by conducting chronoamperometry with a step length of 10 min. Each thin film was tested at least three separate times to ensure statistical relevance of the observed trends. The Faradaic efficiency and partial current density calculation process can be found in Supplementary information.

## Supplementary information

Supplementary Information

Peer Review File

## Data Availability

All the data that support the findings of this study are available within the paper and its supplementary information files, or from the corresponding author on reasonable request.
