# Supplementary material for: Unraveling the rate-limiting step of two-electron transfer electrochemical reduction of carbon dioxide
Source: Nat Commun. 2022 Feb 10;13:803. doi: 10.1038/s41467-022-28436-z (PMC8831479; doi:10.1038/s41467-022-28436-z)
Supplement: Supplementary file 1 — Supplementary Information [file 41467_2022_28436_MOESM1_ESM.pdf]

Supplementary Information

Unraveling the Rate-Limiting Step of Two-Electron Transfer Electrochemical Reduction of Carbon Dioxide

Wanyu Deng[1,2], Peng Zhang[1,3], Brian Seger[2,*], and Jinlong Gong[1,3,*]

Author Address

[1] Key Laboratory for Green Chemical Technology of Ministry of Education, School of Chemical Engineering and Technology, Tianjin University, Collaborative Innovation Center of Chemical Science and Engineering (Tianjin), Tianjin 300072, China

[2] SurfCat, Department of Physics, Technical University of Denmark, 2800 Kgs. Lyngby, Denmark

[3] Joint School of National University of Singapore and Tianjin University, International Campus of Tianjin University, Binhai New City, Fuzhou 350207, China

***Corresponding Author**

E-mail: jlgong@tju.edu.cn.

E-mail: brse@fysik.dtu.dk

The rate expressions and corresponding reaction orders

For an electrochemical reaction (Eqn. S1), the full Butler-Volmer equation (Eqn. S2) can be simplified to Eqn. S3 only if the overpotential is sufficiently high that the rate of the reverse reaction is negligible comparing to that of the forward reaction.

$$O_x + ne^- \rightarrow R_{ed} \quad (S1)$$

$$j = nFk_b^0 a[R_{ed}]\exp[(1-\alpha)f\eta] - nFk_f^0 a[O_x]\exp[-\alpha f\eta] \quad (S2)$$

$$j = -nFk_f^0 a[O_x]\exp(-\alpha f\eta) \quad (S3)$$

In these equations, j is the current density; η is the overpotential for the cathodic reaction; k_f^0 is the standard forward rate constant; k_b^0 is the standard backward rate constant; F is the Faraday constant; $f = F/RT$, where R is the ideal gas constant and T is absolute temperature; α is the transfer coefficient assumed to be equal to 0.5; n is the number of transferred electrons; $a[R_{ed}]$ and $a[O_x]$ are the concentration of reductant and oxidant.

At equilibrium condition ($j = 0$), Eqn. S2 can be simplified to Eqn. S4, and then from a brief deformation Eqn. S5 can be obtained.

$$nFk_b^0 a[R_{ed}]\exp[(1-\alpha)f\eta)] = nFk_f^0 a[O_x]\exp(-\alpha f\eta) \quad (S4)$$

$$a[R_{ed}]/a[O_x] = K^{\theta}\exp(-f\eta) \quad (S5)$$

In these equations, $K^{\theta} = k_f^0/k_b^0$ is the standard equilibrium constant where T = 25 ℃.

For example, for one of the proposed reaction mechanisms as written below (Eqn. S6-S9), the expected rate expression can be derived as a function of reaction intermediates, assuming one of the processes to be the possible rate-limiting step (RLS):

$$D1 \quad CO_2 + * + e^- \rightarrow *CO_2^- \quad (S6)$$

D2 $*CO_2^- + H^+ \rightarrow *OOCH$ (S7)

D3 $*OOCH + e^- \rightarrow *OOCH^-$ (S8)

D4 $*OOCH^- \rightarrow HCOO^- + *$ (S9)

If Eqn. S6 is assumed as the RLS, the following rate expression can be deduced according to Eqn. S3:

$$j_{HCOO^-} = 2Fk_{D1}{}^0 a[CO_2]\theta^* \exp(-\alpha f\eta)$$

If Eqn. S7 is assumed as the RLS, according to Eqn. S3 and Eqn. S5, the following rate expression can be deduced:

$$j_{HCOO^-} = 2Fk_{D2}{}^0 a[*CO_2^-]a[H^+]$$

$$K_{D1}{}^\theta \exp(-f\eta) = a[*CO_2^-]/a[CO_2]\theta^* \Rightarrow a[*CO_2^-] = K_{D1}{}^\theta a[CO_2]\theta^* \exp(-f\eta)$$

$$\Rightarrow j_{HCOO^-} = 2Fk_{D2}{}^0 K_{D1}{}^\theta a[CO_2]\theta^* a[H^+]\exp(-f\eta)$$

If Eqn. S8 is assumed as the RLS, according to Eqn. S3 and Eqn. S5, the following rate expression can be deduced:

$$j_{HCOO^-} = 2Fk_{D3}{}^0 a[*OOCH]\exp(-\alpha f\eta)$$

$$K_{D2}{}^\theta = a[*OOCH]/a[*CO_2^-]a[H^+] \Rightarrow a[*OOCH] = K_{D2}{}^\theta a[*CO_2^-]a[H^+]$$

$$K_{D1}{}^\theta \exp(-f\eta) = a[*CO_2^-]/a[CO_2]\theta^* \Rightarrow a[*CO_2^-] = K_{D1}{}^\theta a[CO_2]\theta^* \exp(-f\eta)$$

$$\Rightarrow a[*OOCH] = K_{D2}{}^\theta K_{D1}{}^\theta a[CO_2]\theta^* a[H^+]\exp(-f\eta)$$

$$\Rightarrow j_{HCOO^-} = 2Fk_{D3}{}^0 K_{D2}{}^\theta K_{D1}{}^\theta a[CO_2]\theta^* a[H^+]\exp(-(1+\alpha)f\eta)$$

If Eqn. S9 is assumed as the RLS, according to Eqn. S3 and Eqn. S5, the following rate expression can be deduced:

$$j_{HCOO^-} = 2Fk_{D4}{}^0 a[*OOCH^-]$$

$$K_{D3}{}^\theta \exp(-f\eta) = a[*OOCH^-]/a[*OOCH] \Rightarrow a[*OOCH^-] = K_{D3}{}^\theta a[*OOCH]\exp(-f\eta)$$

$K_{D2}^{\theta} = a[\text{*OOCH})/a[\text{*CO}_2^-]a[\text{H}^+] \Rightarrow a[\text{*OOCH}] = K_{D2}^{\theta}a[\text{*CO}_2^-]a[\text{H}^+]$

$K_{D1}^{\theta}\exp(-f\eta) = a[\text{*CO}_2^-]/a[\text{CO}_2]\theta^* \Rightarrow a[\text{*CO}_2^-] = K_{D1}^{\theta}a[\text{CO}_2]\theta^*\exp(-f\eta)$

$\Rightarrow a[\text{*OOCH}^-] = K_{D3}^{\theta}K_{D2}^{\theta}K_{D1}^{\theta}a[\text{CO}_2]\theta^*a[\text{H}^+]\exp(-2f\eta)$

$\Rightarrow j_{HCOO^-} = 2Fk_{D4}^{0}K_{D3}^{\theta}K_{D2}^{\theta}K_{D1}^{\theta}a[\text{CO}_2]\theta^*a[\text{H}^+]\exp(-2f\eta)$

In the cases above, the reaction order and Tafel slope can be obtained. By modifying the reduced Butler-Volmer equation to the more common Tafel formula (Eqn. S10)

$$\eta = A + B\log(j) \qquad \text{(S10)},$$

the Tafel slope (B) can be calculated from the rate expression (A is a parameter related to the exchange current density). From the Tafel equation, the Tafel slops for A1, A2, A3, and A4 as the RLSs are 118, 59, 39 and 30 mV·dec^{-1}, respectively. For other possible mechanisms, final results from this analysis are shown in Table 1 and Table 2 in the main text.

The RLS of two-electron transfer CO_2ER can be determined via the analysis of the rate expression and reaction order for different reactants.[1-4] Whether the reaction is controlled by the concentration of H^+ or the reactivity of H_2O molecules can be clarified through pH dependency and kinetic isotope effect (KIE) experiments. However, it is worth pointing out that whether protons are involved in the RLS cannot be simply determined by pH dependency experiments, since H_2O could be the proton source. For example, H_2O would be the proton source for the 'a2' reaction step in Table 1 (Eqn. S11) as shown below

$$\text{*CO}_2^- + \text{H}_2\text{O} \rightarrow \text{*COOH} + \text{OH}^- \qquad \text{(S11)}.$$

An important subtlety that should be noted when CO desorption would be the RLS (A5

from Table 1, Eqn. S12)

$$^*CO \rightarrow {}^* + CO \qquad \text{(S12).}$$

This step itself involves neither H^+ nor H_2O. However, the rate expression (Eqn. S13) actually contains the both since protons take part in the reaction processes before this step.

$$j_{co} = 2Fk^{0}_{A4}K^{\theta}_{A3}K^{\theta}_{A2}K^{\theta}_{A1}a[CO_2]\theta^{*}a^{2}[H^{+}]\exp(-2f\eta)/a[H_2O] \qquad \text{(S13)}$$

Therefore, the above discussions demonstrate the importance of a detailed reaction rate analysis when trying to analyze the RLS.

Faradaic efficiency and partial current density calculation

The Faradaic efficiency (*FE*) of product can be calculated according to the equation:

$$FE(\%) = \frac{Q_{product}}{Q_{tot}} \times 100\% \qquad \text{(S14)}$$

where $Q_{product}$ and Q_{tot} are charge transferred for product formation and charge passed through the working electrode, respectively.

Based on the equation above, the detailed calculation for *FE* of gas product (Eqn. S15) and liquid product (Eqn. S16) could be written as:

$$FE(\%) = \frac{n \times C_{g-product} \times \emptyset \times t \times \frac{P_o}{RT} \times F}{I \times t} \times 100\% \qquad \text{(S15)}$$

$$FE(\%) = \frac{n \times C_{l-product} \times V \times F}{Q_{tot}} \times 100\% \qquad \text{(S16)}$$

where $C_{g\text{-}product}$ and n are the concentration of gas product measured by GC and the number of electrons required for producing one molecule of the related gas product, respectively. $\emptyset$ is gas flow rate and 10 sccm was used for those experiments, t is the electrolysis time which can be deleted in the numerator and denominator, P_o is ambient

pressure, T is absolute temperature (all experiments are performed at ambient temperature, 273.15 K), and I is current. $C_{l\text{-}product}$ is the concentration of liquid product measured by HPLC. V is the liquid value and 6 mL liquid was used as cathodic electrolyte. Q_{tot} is the amount of charge accumulated in 40 minutes.

The j of products can be calculated according to the below equation:

$$j_{product} = \frac{I \times FE_{product}}{S} \tag{S17}$$

Where S is the area of electrode.

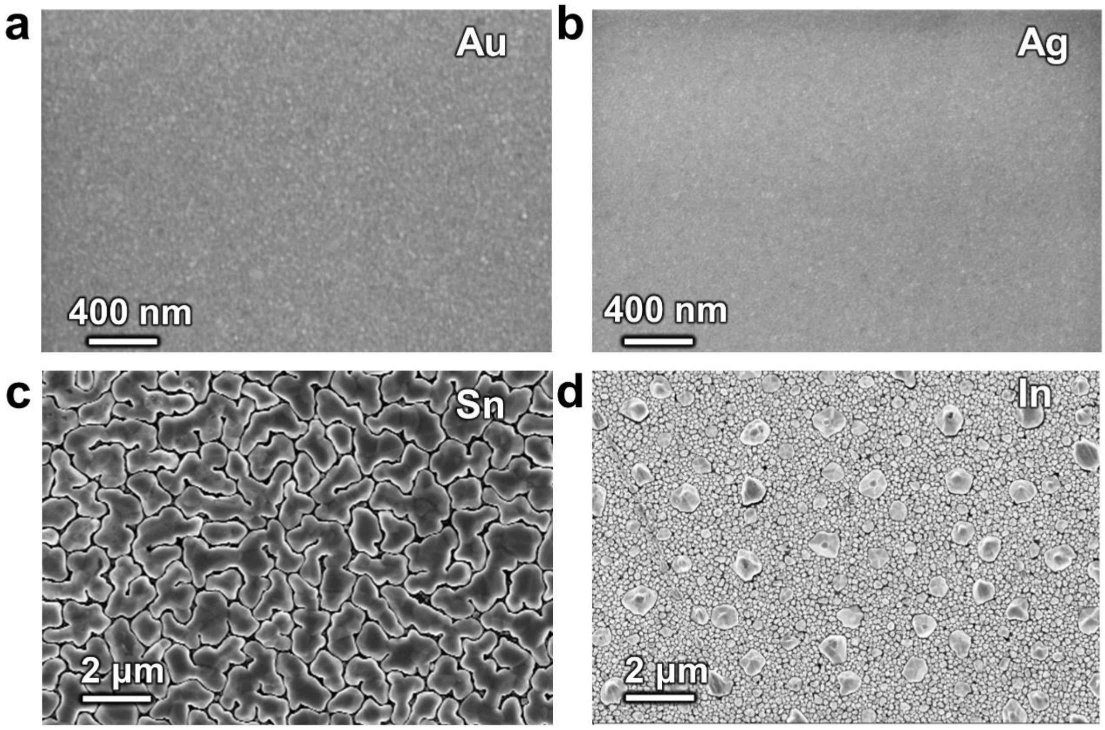

Supplementary Fig. 1. Scanning electron microscopy (SEM) images of the (a) Au film, (b) Ag film, (c) Sn film, and (d) In film.

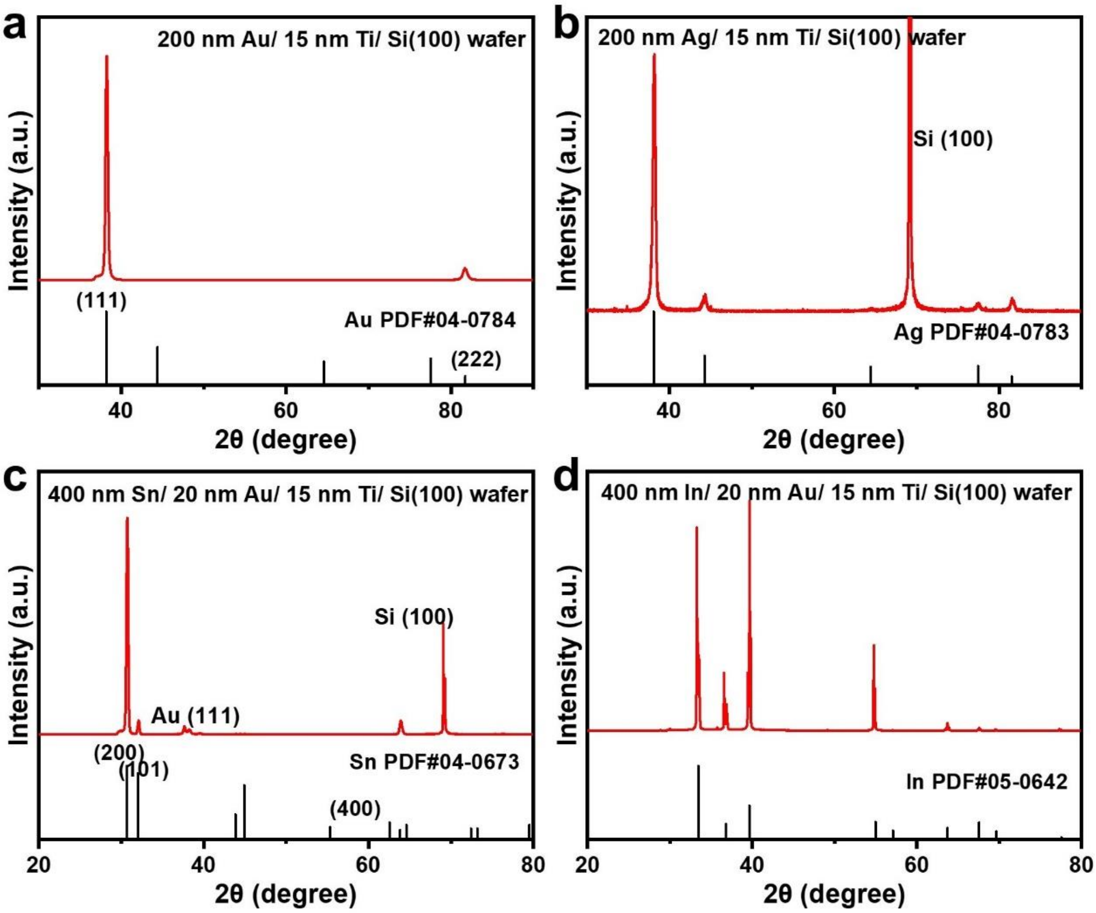

Supplementary Fig. 2. X-ray diffraction (XRD) patterns of the (a) Au film, (b) Ag film, (c) Sn film, and (d) In film.

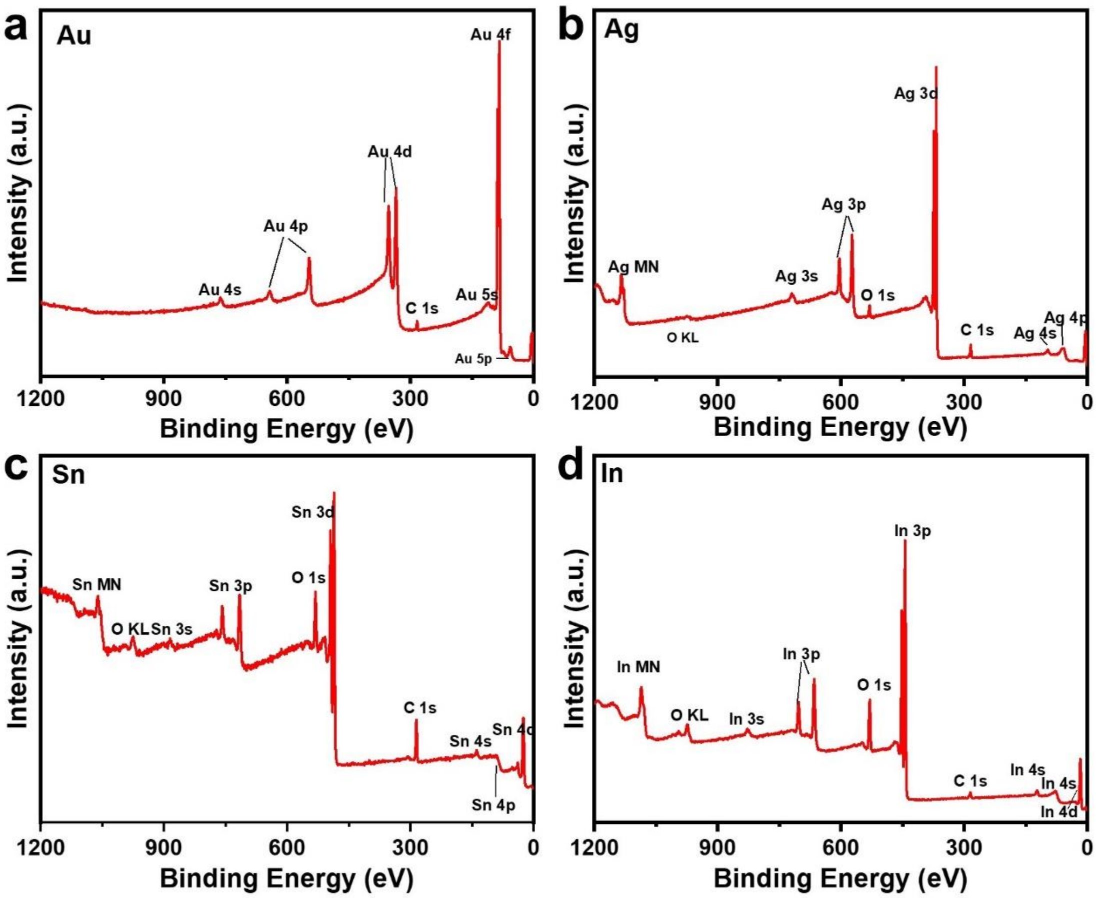

Supplementary Fig. 3. X-ray photoelectron spectroscopy (XPS) spectra in the range of 0-1200 eV for the (a) Au film, (b) Ag film, (c) Sn film, and (d) In film.

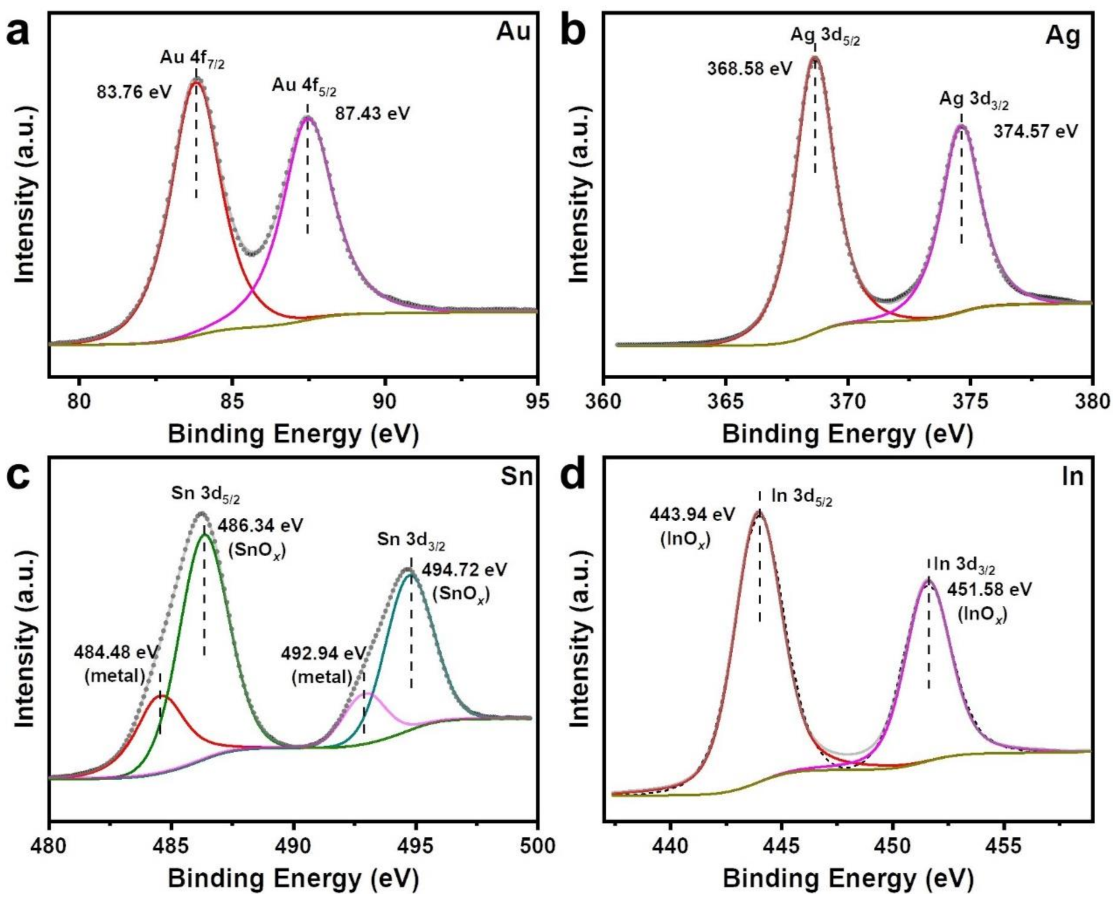

Supplementary Fig. 4. High-resolution XPS spectra and the fitting results in the range of (a) 79-95 eV for the Au film, (b) 361-380 eV for the Ag film, (c) 480-500 eV for the Sn film, and (d) 437-458 eV for the In film.

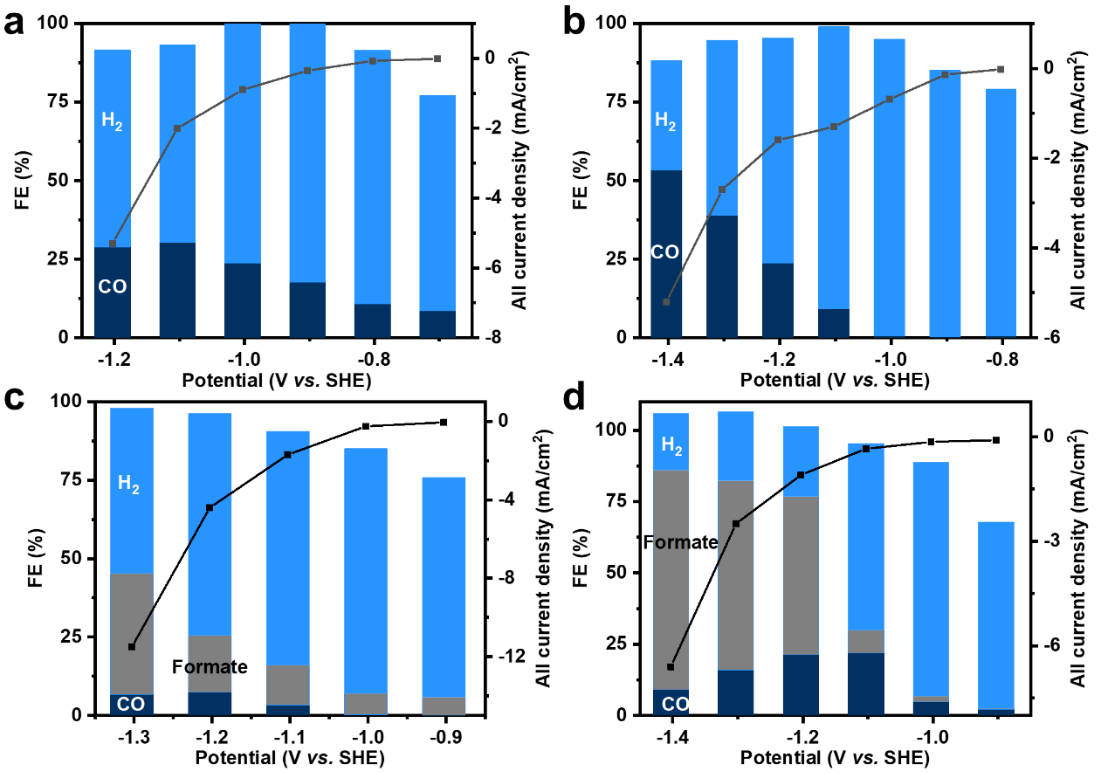

Supplementary Fig. 5. *FE* of products and all current density for Au (a), Ag (b), Sn (c), and In (d) in CO_2-saturated 0.5 M $KHCO_3$.

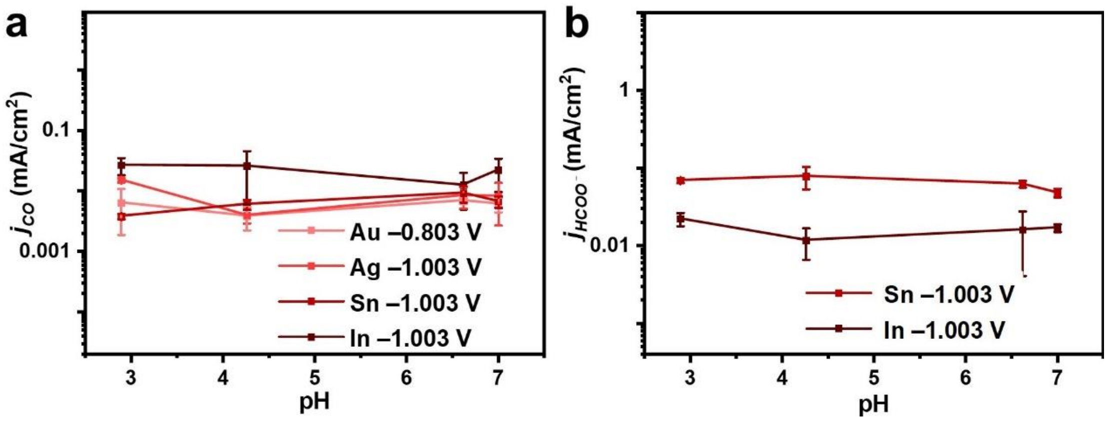

Supplementary Fig. 6. **a**, j_{CO} for Au, Ag, Sn, and In catalysts at fixed potentials of –0.803, –1.003, –1.003 and –1.003 V *vs.* standard hydrogen electrode (SHE), respectively. **b**, j_{HCOO^-} for Sn and In catalysts with at a fixed potential of –1.003 V *vs.* SHE. Error bars are means ± standard deviation (n = 3 replicates)

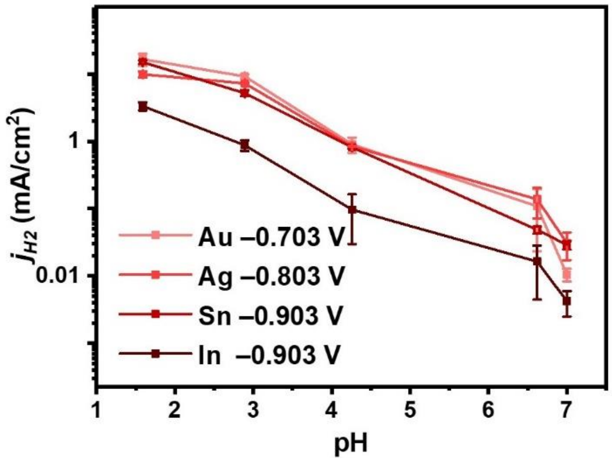

Supplementary Fig. 7. j_{H2} for Au, Ag, Sn, and In catalysts at fixed potentials of –0.703, –0.803, –0.903 and –0.903 V *vs.* SHE, respectively. Error bars are means ± standard deviation (n = 3 replicates)

The local pH calculation

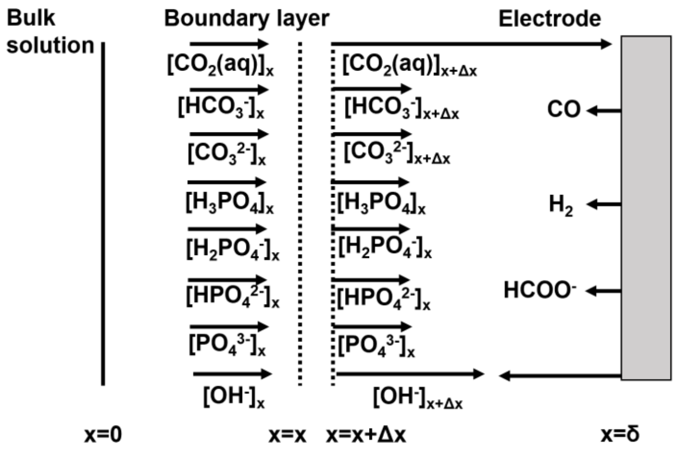

Supplementary Fig. 8. Scheme of states of ions at the electrode-electrolyte boundary layer.

According to M. Gattrell's method[5], Supplementary Fig. 8 shows the states of ions at the electrode-electrolyte boundary layer in the CO_2 electroreduction system. Film theory is assumed to be applicable where, in the concentration boundary layer, the velocity gradients or convective effects are assumed to be negligible. According to the equilibrium reaction[6]:

$$CO_2(aq)+OH^- \Leftrightarrow HCO_3^- \quad K_1=4.44*10^7\ (k_{1f}= 5.93*10^3;\ k_{1b}= 1.34*10^{-4}) \tag{S18}$$

$$HCO_3^-+OH^- \Leftrightarrow CO_3^{2-}+H_2O \quad K_2=4.66*10^3\ (k_{2f}= 1*10^8;\ k_{2b}= 2.15*10^4) \tag{S19}$$

$$H_2PO_4^-+H_2O \Leftrightarrow H_3PO_4+OH^- \quad K_3=1.41*10^{-12}\ (k_{3f}= 1*10^2;\ k_{3b}= 7.09*10^{13}) \tag{S20}$$

$$HPO_4^{2-}+H_2O \Leftrightarrow H_2PO_4^-+OH^- \quad K_4=1.58*10^{-7}\ (k_{4f}= 1*10^4;\ k_{4b}= 6.33*10^{10}) \tag{S21}$$

$$PO_4^{3-}+H_2O \Leftrightarrow HPO_4^{2-}+OH^- \quad K_4=2.09*10^{-2}\ (k_{4f}= 1.05*10^7;\ k_{4b}= 5.02*10^8) \tag{S22}$$

The following balances would then occur within a slice of solution from x to x+ Δ x in Supplementary Fig. 8:

$$\frac{\partial[CO_2(aq)]}{\partial t}=D_{CO_2}\frac{\partial^2[CO_2(aq)]}{\partial x^2}-k_{1f}[CO_2(aq)][OH^-]+k_{1b}[HCO_3^-] \tag{S23}$$

$$\frac{\partial[HCO_3^-]}{\partial t}=D_{HCO_3^-}\frac{\partial^2[HCO_3^-]}{\partial x^2}+k_{1f}[CO_2(aq)][OH^-]-k_{1b}[HCO_3^-]-k_{2f}[HCO_3^-][OH^-]+k_{2b}[CO_3^{2-}] \quad (S24)$$

$$\frac{\partial[CO_3^{2-}]}{\partial t}=D_{CO_3^{2-}}\frac{\partial^2[CO_3^{2-}]}{\partial x^2}+k_{2f}[HCO_3^-][OH^-]-k_{2b}[CO_3^{2-}] \quad (S25)$$

$$\frac{\partial[H_3PO_4]}{\partial t}=D_{H_3PO_4}\frac{\partial^2[H_3PO_4]}{\partial x^2}+k_{3f}[H_2PO_4^-]-k_{3b}[H_3PO_4][OH^-] \quad (S26)$$

$$\frac{\partial[H_2PO_4^-]}{\partial t}=D_{H_2PO_4^-}\frac{\partial^2[H_2PO_4^-]}{\partial x^2}+k_{4f}[HPO_4^{2-}]-k_{4b}[H_2PO_4^-][OH^-]-k_{3f}[H_2PO_4^-]+k_{3b}[H_3PO_4][OH^-] \quad (S27)$$

$$\frac{\partial[HPO_4^{2-}]}{\partial t}=D_{HPO_4^{2-}}\frac{\partial^2[HPO_4^{2-}]}{\partial x^2}+k_{5f}[PO_4^{3-}]-k_{5b}[HPO_4^{2-}][OH^-]-k_{4f}[HPO_4^{2-}]+k_{4b}[H_2PO_4^-][OH^-] \quad (S28)$$

$$\frac{\partial[PO_4^{3-}]}{\partial t}=D_{PO_4^{3-}}\frac{\partial^2[PO_4^{3-}]}{\partial x^2}-k_{5f}[PO_4^{3-}]+k_{5b}[HPO_4^{2-}][OH^-] \quad (S29)$$

$$\frac{\partial[OH^-]}{\partial t}=D_{OH^-}\frac{\partial^2[OH^-]}{\partial x^2}-k_{1f}[CO_2(aq)][OH^-]+k_{1b}[HCO_3^-]-$$

$$k_{2f}[HCO_3^-][OH^-]+k_{2b}[CO_3^{2-}]+k_{3f}[H_2PO_4^-]- \quad k_{3b}[H_3PO_4][OH^-]+k_{4f}[HPO_4^{2-}]-k_{4b}[H_2PO_4^-][OH^-]+k_{5f}[PO_4^{3-}]-$$

$$k_{5b}[HPO_4^-][OH^-] \quad (S30)$$

The diffusion coefficients at infinite dilution in water for various species at 25 ° C are listed in Supplementary Table 1.

Supplementary Table 1. Diffusion coefficients (10^{-10} D/ m^2/s) at 25 ° C in water.[7-9]

$CO_2(aq)$	HCO_3^-	CO_3^{2-}	H_3PO_4	$H_2PO_4^-$	HPO_4^{2-}	PO_4^{3-}	OH^-
19.1	9.23	11.9	9.10	9.58	7.59	7.00	52.7

CO_2 is reduced to a variety of products with different current efficiencies according to overall reactions Eqn. S31 to Eqn. S32. Also, water could be reduced to H_2 according to Eqn. S33.

$$CO_2(aq)+H_2O+2e^- \rightarrow HCOO^-+OH^- \quad (S31)$$

$$CO_2(aq)+H_2O+2e^- \rightarrow CO+2OH^- \quad (S32)$$

$$H_2O+2e^- \rightarrow H_2+2OH^- \quad (S33)$$

Based on these reactions, the boundary conditions at time t > 0 and x = δ (i.e., at the electrode surface) are related to the reaction fluxes:

$$D_{CO_2}\frac{\partial[CO_2(aq)]}{\partial x}=\frac{j}{F}\times\left(\frac{FE_{CO}}{2}+\frac{FE_{HCOO^-}}{2}\right)$$

(S34)

$$D_{HCO_3^-}\frac{\partial[HCO_3^-]}{\partial x}=0 \quad (S35)$$

$$D_{H_3PO_4}\frac{\partial[H_3PO_4]}{\partial x}=0 \quad (S36)$$

$$D_{H_2PO_4^-}\frac{\partial[H_2PO_4^-]}{\partial x}=0 \quad (S37)$$

$$D_{HPO_4^{2-}}\frac{\partial[HPO_4^{2-}]}{\partial x}=0 \quad (S38)$$

$$D_{PO_4^{3-}}\frac{\partial[PO_4^{3-}]}{\partial x}=0 \quad (S39)$$

$$D_{CO_3^{2-}}\frac{\partial[CO_3^{2-}]}{\partial x}=0 \quad (S40)$$

$$D_{OH^-}\frac{\partial[OH^-]}{\partial x}=\frac{j}{F}\times\left(2\times\frac{FE_{CO}}{2}+\frac{FE_{HCOO^-}}{2}+2\times\frac{FE_{H_2}}{2}\right) \quad (S41)$$

The boundary layer is assumed to be 8 μm. The initial values of the concentrations (at t=0, before current flows) are assumed to be the same as those of the bulk solution and are listed in Supplementary Table 2 for different electrolytes. At time t > 0 and x = 0 (i.e., at the interface of bulk solution and the boundary layer) the boundary condition are the equilibrium values in the bulk solution, which are the same as the values given in Supplementary Table 2.

Supplementary Table 2. Initial equilibrium values (t = 0) at different electrolytes at 25 ° C and 101.3 kPa CO_2 partial pressure.

Electrolyte	$CO_2(aq)$	HCO_3^-	CO_3^{2-}	H_3PO_4	$H_2PO_4^-$	HPO_4^{2-}	PO_4^{3-}	OH^-
0.3 M $KHCO_3$	3.42×10^{-2}	3.00×10^{-1}	2.76×10^{-4}					2.00×10^{-7}
0.1 M K_3PO_4	3.42×10^{-2}	6.33×10^{-2}	1.26×10^{-5}	2.52×10^{-6}	7.95×10^{-2}	2.04×10^{-2}	8.40×10^{-8}	4.17×10^{-8}

0.1 M KH_2PO_4	3.42×10^{-2}	2.76×10^{-4}	2.34×10^{-10}	7.70×10^{-4}	9.91×10^{-2}	1.14×10^{-2}	1.00×10^{-7}	1.82×10^{-10}
0.1 M KH_2PO_4 with H_3PO_4	3.42×10^{-2}	1.18×10^{-5}	4.36×10^{-13}	1.45×10^{-2}	8.55×10^{-2}	4.09×10^{-6}	9.00×10^{-9}	7.76×10^{-12}
0.1 M H_3PO_4	3.42×10^{-2}	5.91×10^{-7}	1.10×10^{-15}	7.72×10^{-2}	2.28×10^{-2}	5.47×10^{-8}	4.53×10^{-8}	3.89×10^{-13}

With all the boundary conditions and constants known and the reaction current density of In film, the partial differential equations given in Eqn. S23-Eqn. S30 were solved using MATLAB R2013b.

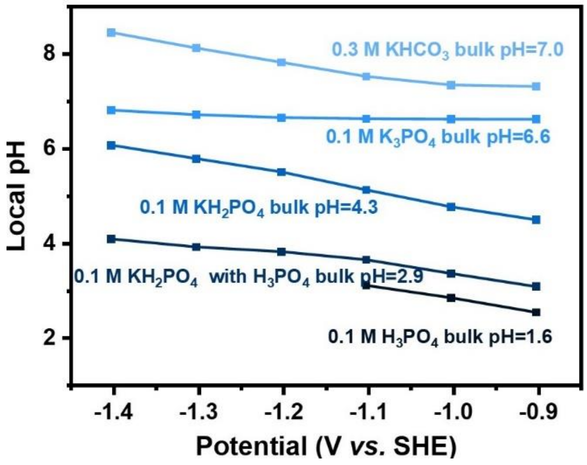

Supplementary Fig. 9. Calculated local pH in different electrolytes during CO_2ER for In catalyst. The thickness of the boundary layer is assumed to be 8 μm.

The degree to which the local pH varies from the bulk pH is dependent on the rate at which the reaction byproduct OH^- can diffuse away from the catalyst. Thus, the local pH is defined experimentally by the reaction rate (i.e., current density) and the boundary layer thickness. The same reactor was used for all experiments thus the boundary layer will always be the same (8 μm), and all catalysts also operated roughly within the same current density range (Fig. 1-3). These two conditions entail that local pH variations will be similar for most catalysts, and thus by analyzing just one of the catalysts we can get a good understanding of local pH issues for all catalysts. In was therefore taken as a representative catalyst. Supplementary Fig. 9 shows the local pH as a function of the operating potential for In, where the local pH elevates slowly with an increasing reaction potential (so as to current density) for those electrolytes, but the overall trends are the same. The similar potential dependency of the local pH demonstrates that it is reasonable to substitute bulk pH for local pH for the current work.

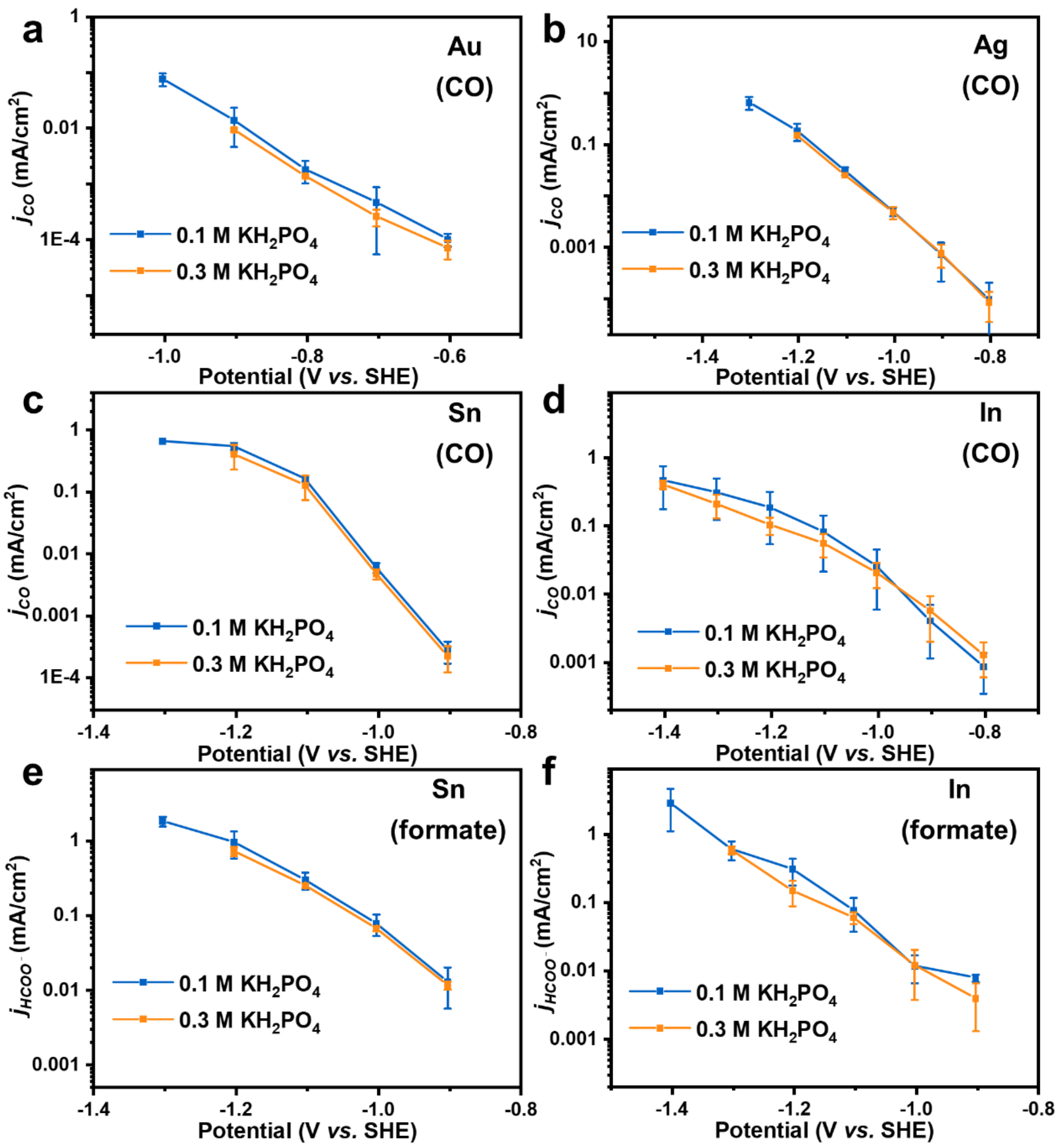

Supplementary Fig. 10. a-d, The j_{CO} for Au (**a**), Ag (**b**), Sn (**c**), and In (**d**) catalysts in CO_2-saturated 0.1 and 0.3 M KH_2PO_4. **e**,**f**, The j_{HCOO^-} for Sn (**e**) and In (**f**) catalysts in CO_2-saturated 0.1 and 0.3 M KH_2PO_4. Error bars are means ± standard deviation (n = 3 replicates)

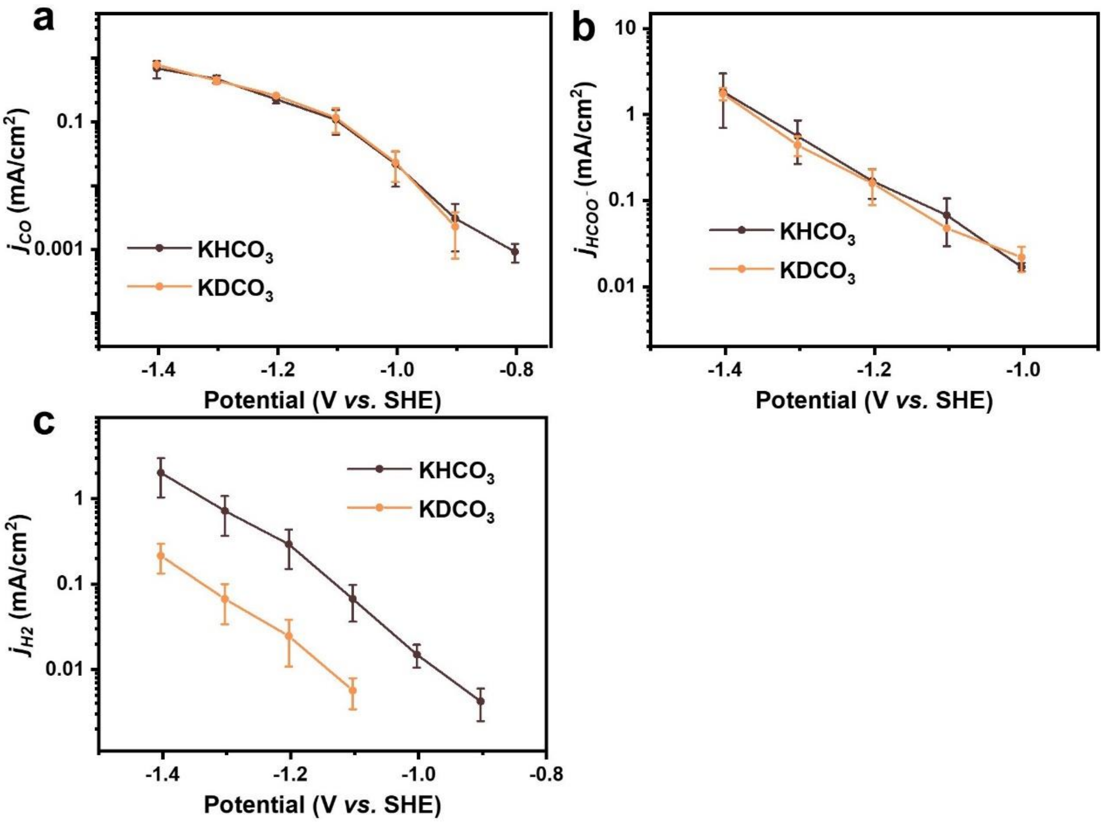

Supplementary Fig. 11. (a) j_{CO}, (b) j_{HCOO^-}, and (c) j_{H2} for In catalyst at 0.3 M CO_2 saturated $KHCO_3$ and $KDCO_3$ electrolytes, which are obtained by bubbling CO_2 into 0.15 M K_2CO_3 H_2O and D_2O solutions overnight. Error bars are means ± standard deviation (n = 3 replicates)

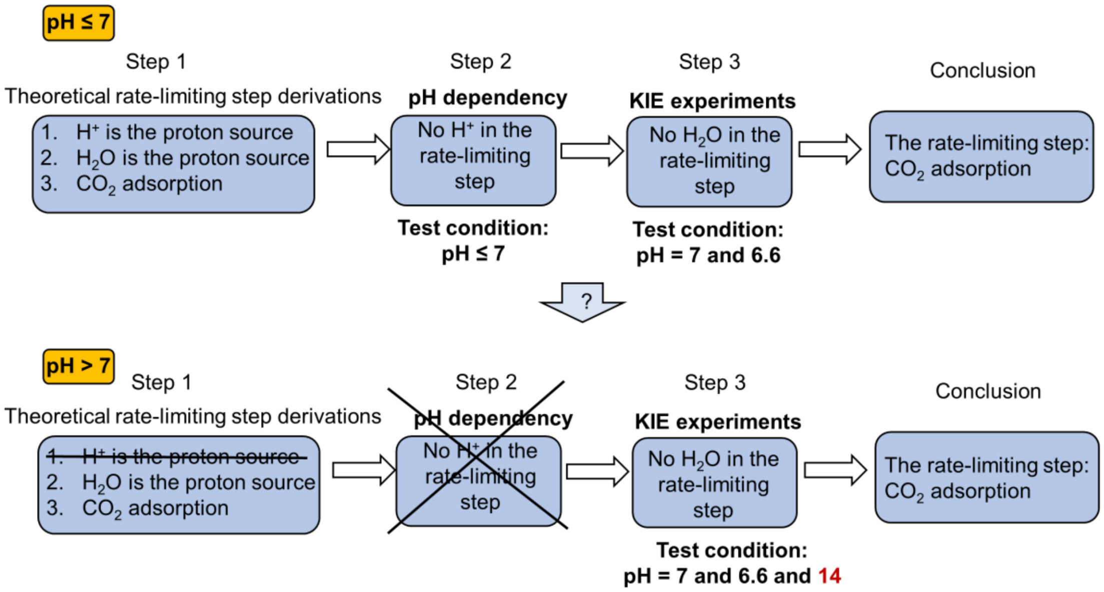

Supplementary Fig. 12. The proof for the conclusion in this article.

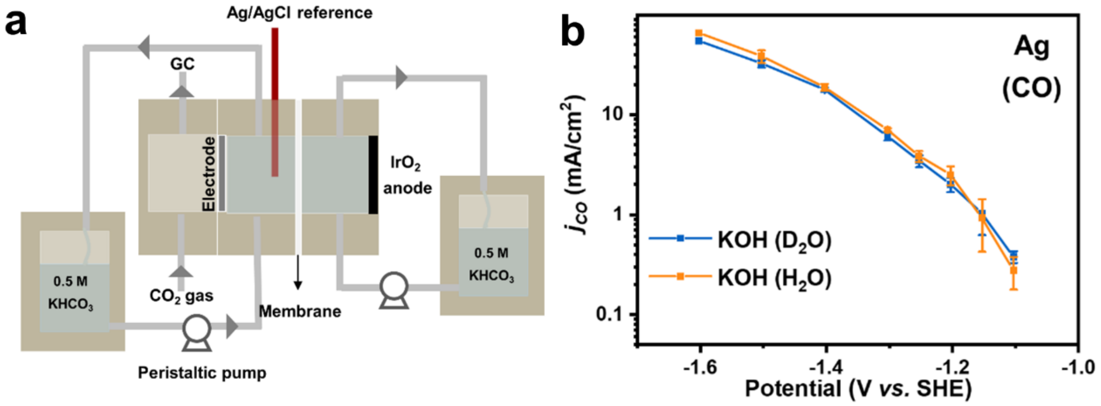

Supplementary Fig. 13. a. The diagram of flow cell. **b**. The CO current density of Ag in flow cell under 0.3 M KOH electrolyte with H_2O or D_2O. Error bars are means ± standard deviation (n = 3 replicates)

In this paper, the conclusion based on the pH dependency and KIE experiments can be obtained (Supplementary Fig. 12). To prove whether the conclusion is applicable to alkaline solutions, it is necessary to go through all proof steps. For step 1, the difference between alkaline and acidic solutions is that H^+ is not considered as the source of protons under alkaline solutions,[10,11] so the step2 become unnecessary. Now, the problem is that whether the conclusion of step 3 is applicable to alkaline solutions. Here, for the KIE experiments conducted under neutral solutions, it seems reasonable to extend the conclusion that H_2O is not involved in the RLS from neutral solutions to alkaline solutions. Because, under both electrolytes, the difference is the concentration of OH-, while the concentration of H_2O is unchanged. To be more accurate, the KIE experiments under alkaline solutions in the flow cell (Supplementary Fig. 13a) was conducted to avoid the dissolution of CO_2 in the solution as much as possible. For Ag,

the CO current density is basically the same in 0.3 M KOH electrolyte with H_2O or D_2O (Supplementary Fig. 13b), which means that the conclusion of the KIE experiment will not be affected by the alkaline solutions.

Supplementary Table 3. The R_u for different catalysts in different electrolytes.

Electrolyte	Au /Ω	Ag /Ω	Sn /Ω	In /Ω
0.3 M $KHCO_3$	23	23	25	26
0.1 M K_3PO_4	41	39	39	41
0.1 M KH_2PO_4	66	63	74	75
0.1 M KH_2PO_3 with H_3PO_4	79	68	83	81
0.1 M H_3PO_4	63	61	77	80
0.1 M KH_2PO_4 (D_2O)	72	74	92	90
0.3 M $KDCO_3$ (D_2O)	-	-	-	33
0.3 M KH_2PO_4	29	30	33	32

The potentiostat compensated for 85% of R_u in situ and the last 15% was post corrected to arrive at accurate potentials since the potentiostat cannot give 100% potentials correction:[12]

$$E(\text{Real potential}) = E(\text{Applied potential}) - I(\text{Current}) \times R_u \times 0.15 \qquad \text{(S42)}.$$

All the R_u for different catalysts in different electrolytes is listed in Supplementary Table 3.

Supplementary References

1. Varela, A. S., *et al.* pH effects on the selectivity of the electrocatalytic CO_2 reduction on graphene-embedded Fe–N–C motifs: bridging concepts between molecular homogeneous and solid-state heterogeneous catalysis. *ACS Energy Lett.* **3**, 812-817 (2018).
2. Ringe, S., *et al.* Double layer charging driven carbon dioxide adsorption limits the rate of electrochemical carbon dioxide reduction on gold. *Nat. Commun.* **11**, 33 (2020).
3. Strmcnik, D., *et al.* Improving the hydrogen oxidation reaction rate by promotion of hydroxyl adsorption. *Nat. Chem.* **5**, 300-306 (2013).
4. Vijay, S., Gauthier, J. A., Heenen, H. H., Bukas, V. J., Kristoffersen, H. H. & Chan, K. Dipole-field interactions determine the CO_2 reduction activity of 2D Fe–N–C single-atom catalysts. *ACS Catal.* **10**, 7826-7835 (2020).
5. Gupta, N., Gattrell, M. & MacDougall, B. Calculation for the cathode surface concentrations in the electrochemical reduction of CO_2 in $KHCO_3$ solutions. *J. Appl. Electrochem.* **36**, 161-172 (2005).
6. Yasunaga, T., Tanoura, M. & Miura, M. Ultrasonic investigation on kinetics of ionic reaction in sodium phosphate solution. *J. Chem. Phys.* **43**, 3512-3518 (1965).
7. Lang, S., Kazdal, T. J., Kühl, F. & Hampe, M. J. Diffusion coefficients and VLE data of aqueous phosphoric acid. *J. Chem. Phys.* **68**, 75-81 (2014).
8. Pismenskaya, N., Sarapulova, V., Nevakshenova, E., Kononenko, N., Fomenko, M. & Nikonenko, V. Concentration dependencies of diffusion permeability of anion-exchange membranes in sodium hydrogen carbonate, monosodium phosphate, and potassium hydrogen tartrate solutions. *Membranes (Basel)* **9**, 170 (2019).
9. Sullivan, B. P., Krist, K. & Guard, H. *Electrochemical and electrocatalytic reactions of carbon dioxide*. Elsevier (2012).
10. Ooka, H., Figueiredo, M. C. & Koper, M. T. M. Competition between hydrogen evolution and carbon dioxide reduction on copper electrodes in mildly acidic media. *Langmuir* **33**, 9307-9313 (2017).
11. Mahmood, N., Yao, Y., Zhang, J.-W., Pan, L., Zhang, X. & Zou, J.-J. Electrocatalysts for hydrogen evolution in alkaline electrolytes: mechanisms, challenges, and prospective solutions. *Adv. Sci.* **5**, 1700464 (2018).
12. Ringe, S., *et al.* Understanding cation effects in electrochemical CO_2 reduction. *Energy Environ. Sci.* **12**, 3001-3014 (2019).
